# Anthrax outbreaks in the humans - livestock and wildlife interface areas of Northern Tanzania: a retrospective record review 2006–2016

**DOI:** 10.1186/s12889-017-5007-z

**Published:** 2018-01-05

**Authors:** Elibariki Reuben Mwakapeje, Sol Høgset, Robert Fyumagwa, Hezron Emmanuel Nonga, Robinson Hammerthon Mdegela, Eystein Skjerve

**Affiliations:** 1 Epidemiology and Diseases Control Section, Ministry of Health, Community Development, Gender, Elderly and Children, P. O. Box. 9083, Dar es Salaam, Tanzania; 2 Tanzania Wildlife Research Institute (TAWIRI), P. O. Box. 661, Arusha, Tanzania; 3 Department of Veterinary Medicine and Public Health, Sokoine University of Agriculture, P. O. Box. 3021, Chuo Kikuu Morogoro, Tanzania; 4 Faculty of Veterinary Medicine, Norwegian University of Life Sciences, P. O. Box. 8146 Dep., N –, 0033 Oslo, Norway

**Keywords:** Anthrax outbreaks, Wildlife interface areas, Record review, Northern Tanzania

## Abstract

**Background:**

Anthrax outbreaks in Tanzania have been reported from the human, livestock and wildlife sectors over several years, and is among the notifiable diseases. Despite frequent anthrax outbreaks, there is no comprehensive dataset indicating the magnitude and distribution of the disease in susceptible species. This study is a retrospective review of anthrax outbreaks from the human, livestock, and wildlife surveillance systems from 2006 to 2016. The objectives were to identify hotspot districts, describe anthrax epidemiology in the hotspot areas, evaluate the efficiency of the anthrax response systems and identify potential areas for further observational studies.

**Methods:**

We prepared a spreadsheet template for a retrospective comprehensive record review at different surveillance levels in Tanzania. We captured data elements including demographic characteristics of different species, the name of health facility, and date of anthrax diagnosis. Also, we collected data on the date of specimen collection, species screened, type of laboratory test, laboratory results and the outcome recorded at the end of treatment in humans. After establishing the database, we produced maps in Quantum GIS software and transferred cleaned data to Stata software for supportive statistical analysis.

**Results:**

Anthrax reported incidences over 4 years in humans were much higher in the Arusha region (7.88/100,000) followed by Kilimanjaro region (6.64/100,000) than other regions of Tanzania Mainland. The health facility based review from hotspot districts in parts of Arusha and Kilimanjaro regions from 2006 to 2016, identified 330 human anthrax cases from the selected health facilities in the two regions. Out of 161 livestock and 57 wildlife specimen tested, 103 and 18 respectively, were positive for anthrax.

**Conclusion:**

This study revealed that there is gross under-reporting in the existing surveillance systems which is an obstacle for estimating a true burden of anthrax in the hotspot districts. Repeated occurrences of anthrax in livestock, wildlife and humans in the same locations at the same time calls for the need to strengthen links and promote inter–disciplinary and multi-sectoral collaboration to enhance prevention and control measures under a One Health approach.

## Background

Anthrax is a zoonotic infectious disease caused by *Bacillus anthracis*, a spore-forming, Gram-positive bacterium [1]. The disease occurs in humans, and wild and domestic mammalian species, in particular, herbivores [2]. Anthrax cases in humans are classified into three forms according to the clinical features and transmission routes: the cutaneous form, accounting for about 95% of all reported human cases worldwide, the gastrointestinal form, and the pulmonary form [3]. There is no evidence of person-to-person transmission of *B. anthracis* [4], and humans normally acquire the disease from direct contact with anthrax-infected animals or anthrax-contaminated animal products [4, 5]. Both domestic and wild animals serve as potential sources of infections in humans [6, 7]. The clinical presentation of this disease in susceptible herbivores is usually characterised by septicaemia and sudden death with/without bleeding from natural orifices and subcutaneous haemorrhages. Other symptoms in livestock and some wild herbivores are fever, dyspnoea, agitation, convulsions followed by sudden death. In pigs, carnivores, and primates the main symptoms are local oedema and swelling of the face and neck. Failure of the blood to clot, the absence of rigor mortis and the presence of splenomegaly are the most significant necropsy findings [6].

Worldwide, anthrax occurs at a low incidence in developed countries but remains endemic in African and Asian regions [6]. The African experience also illustrates the classic One Health aspects of anthrax where humans, livestock, wildlife and environment are important part of the epidemiological pattern. Anthrax outbreaks in Tanzania have been reported in humans, livestock, and wildlife over several years, and areas mostly affected are those in the livestock-wildlife interface [7]. Anthrax outbreaks in hippos were reported in Ugalla Game Reserve in 2000 and 2001 as well as in Mtera dam in 2003 [4]. Several large outbreaks (> 500 deaths) have also been reported in cattle, goats, and sheep in the eastern part of the Serengeti National Park [7]. In September 2016, a total of 153 hippopotamus died in Kilombero River due to anthrax outbreak, and in early October 2016, an anthrax outbreak in livestock occurred in Ngorongoro district, where ten humans were infected, two of them died [8]. Anthrax outbreaks have been reported in the Serengeti ecosystem for many years, mostly with sporadic outbreaks in several endemic hotspot areas affecting humans, livestock and wildlife animals [9].

Anthrax incidence in a given locality is related to temperature, rains or drought, soil, vegetation, host condition and population density [10, 11]. The local weather condition of an area may directly or indirectly influence possibilities for animals to come into contact with *B. anthracis* spores. This may include grazing closer to the soil in dry periods when grasses are short or sparse, and movement of herds to protected areas for wildlife conservation when water becomes scarce. The general state of health of the hosts may also affect their level of resistance to infection [6].

The health personnel in the Ministries responsible for health of human, livestock and wildlife (Epidemiology Unit) and the Ministry of Regional Administration and Local Government are responsible for responding to disease outbreaks as soon as they get outbreak notification from lower levels. During the disease outbreak response, their role is to identify and characterize the outbreak etiologic agents, monitoring the progress of the outbreak and putting the effectiveness of control and preventive strategies in place [12]. From the national level, information of public health emergence or disease outbreak is required to be communicated to WHO within 24 h [13, 14]. For the human surveillance system, communication during surveillance, reporting, and the response is by telephones (mobile and landlines), internet, fax, radio (national and local stations), television, letters, technical Meetings (National task force) and workshops [15]. Laboratory diagnostic reports of anthrax from Tanzania Veterinary Laboratory Agency (TVLA) and Tanzania Wildlife Research Institute (TAWIRI) Serengeti are regularly shared by the Ministries responsible for livestock and wildlife respectively. These reports are crucial for setting up control measures of anthrax outbreaks in livestock and wildlife.

Regular analysis of diagnostic and surveillance data from livestock and wildlife are essential for efficient management of anthrax outbreaks in animals and protecting human population [16].

However, despite the frequent occurrence of anthrax outbreaks in Tanzania, there is no comprehensive analysis of data indicating the magnitude and spatial distribution of the disease from the human, livestock and wildlife health sectors. It is therefore important to coalesce and summarize the available information to assess more comprehensive epidemiological patterns of anthrax in Tanzania.

We conducted a retrospective review of reported anthrax outbreak records from the human, livestock, and wildlife surveillance systems of Tanzania from January 2013 to December 2016. This was followed by a more thorough examination of data from Arusha and Kilimanjaro regions for 2006 to 2016. The specific objectives were (i) to identify the districts assumed to be an anthrax hotspots in Arusha and Kilimanjaro regions, (ii) to evaluate the efficiency of the anthrax reporting and response system and diagnostic capacity at national, regional and district levels, (iii) to describe the epidemiology of anthrax in the hotspot areas and (iv) to identify potential areas for further observational studies to better understand the complex ecology of anthrax.

## Methods

### Study areas

A follow – up was done at national level involving the Ministries responsible for health of humans, livestock and wildlife using a structured checklist. During this follow up, all regions of the Tanzania Mainland were assessed for the described anthrax outbreaks during the period of 2013–2016. After compiling the National data for humans and livestock, we focused on the identified hotspot areas of **Arusha** and **Kilimanjaro** regions for more detailed studies of anthrax data for humans and livestock as well as wildlife.

**Arusha region** lies on the Kenyan border, encompassing savannahs and part of the Great Rift Valley. It has a total area of 37,576 km^2^ with a human population of 1.7 million [17]. Wildlife conservation areas in this region include (a) the Ngorongoro Conservation Area, which contains the Ngorongoro Crater, (b) Arusha National Park, which covers volcanic Mount Meru, (c) Loliondo Game Controlled Area and (d) Lake Natron Game Controlled Area, which contains the active volcanic mountain - Oldoinyo Lengai. This region has seven districts which are Arusha City, Arusha rural, Meru, Ngorongoro, Karatu, Monduli and Longido districts. Based on the history of frequent anthrax outbreaks, a data review was purposively done in Ngorongoro, Meru and Monduli districts for the period of 2006 to 2016.

**Kilimanjaro region** is a home to the highest mountain in Africa, Mt. Kilimanjaro and Kilimanjaro National Park. It is bordered to the north and east by Kenya, to the south by Tanga region, to the southwest by Manyara region and the west by Arusha Region, and has a total area of 13,250 km2 with a population of approximately 1.6 million [17]. The region has seven districts: Hai, Moshi rural, Rombo, Mwanga, Siha, and Same districts, and Moshi Municipality. Hai, Siha, Moshi rural and Rombo districts were conveniently selected for a comprehensive retrospective data review for anthrax outbreaks in the period of 2006 to 2016.

### The National Anthrax Surveillance Systems

Anthrax is among the notifiable diseases in humans in Tanzania and is therefore also included in the current human health electronic integrated diseases surveillance and response system (eIDSR) [14]. Under the described surveillance system, a registered mobile phone is used to report a human suspected anthrax case within 24 h after having met the standard case definition for anthrax at a reporting health facility. All health facilities, Points of Entry (PoE) and any other location (in conjunction with a nearby community) must report the total number of human cases and deaths seen in a given period.

Anthrax is also a notifiable disease in livestock and wildlife, and surveillance systems linked to farms, laboratories, clinics, livestock markets, slaughterhouses and dip tanks are among the data sources for animal health information system (AHIS). More than 80% of disease information obtained is based on clinical observations, and 95% of the surveillance system is paper based investigation, surveillance and treatment reports.

The animal health system is composed of community animal health service under the public sector, and animal health care centres and clinics under the private sector [15]. In wildlife, anthrax reports are submitted through the Veterinary Section at (TAWIRI), where the occurrence of any disease (outbreak, infectious, zoonotic, unknown) is reported to the Director of Veterinary Services. The laboratory personnel working within the wildlife health system include laboratory attendants, laboratory technicians and laboratory technologists [15].

All final human and animal anthrax reports are made available from the Ministries responsible for the health of humans, livestock, and wildlife.

### Standard case definitions

In our follow-up we defined a human anthrax case as follows:

**At the health facility level**, a suspect human anthrax case was any person with acute onset of illness characterized by one of several clinical forms:

1. **Localized form**
  Cutaneous; skin lessions evolving from a papular through a vesicular stage, to a depressed black scar invariably accompanied by oedema that may be mild or extended.
2. **Systemic form**
  - Gastrointestinal; any person with abdominal distress characterized by nausea, hematemesis, blood dirrhoea, vomiting, anorexia and followed by fever
  - Pulmonary; anyone with an acute illness resembling a viral respiratory illness followed by hypoxia, dyspnea or acute respiratory distress with resulting cyanosis and shock.
  - Meningeal; any person with acute illness revealing fever, convulsions, coma, or meningeal signs.

**At the community level, a suspect anthrax was** anyone with fever, difficulty in breathing, skin conditions or abdominal pain or altered consciousness, with a history of contact with sick or dead animal [14].

We defined a suspect anthrax case in a non-immunized livestock or wildlife animal as an acute disease characterised by septicaemia and/or sudden death with/without bleeding from natural orifices and/or could include subcutaneous hemorrhages. Other symptoms in cattle, horses, sheep and some wild herbivores are fever, dyspnoea, agitation, convulsions followed by sudden death. In pigs, carnivores, and non human primates symptoms could include local oedema, and/or swelling of the face and neck. Necropsy, if completed, could reveal failure of the blood to clot, the absence of rigor mortis, and/or the presence of splenomegaly [6].

**Confirmed case**: A suspect case with one of the following:

- Culture and identification of *B. anthracis* from clinical specimens by the designated laboratory or demonstration of *B. anthracis* antigens in tissues by immunohistochemically staining.
- A four-fold increase or change in antibodies to protective antigen between acute and/or paired convalescent sera
- Evidence of *B. anthracis* DNA in blood, swab or tissue specimens collected from a normally sterile site or lesion of other affected tissue (skin, pulmonary, reticuloendothelial, or gastrointestinal).

### National anthrax records review

Sources of data used in this review were the various Epidemiology sections of the Ministries responsible for human and livestock services.We examined nationally stored databases of each Ministry to retrieve the relevant surveillance data for the period of January 2013 to December 2016. The Ministry of Natural Resources and Tourism (MNRT) headquarters receives the active surveillance reports from TAWIRI which prepares reports after every wildlife related outbreak occurring in the protected areas of Tanzania. Therefore, we did a record review for 2006 to 2016 at the TAWIRI research centre located in the Serengeti National Park. We also compiled information about the population structure of humans and livestock based upon the statistics obtained from the National Bureau of Statisticts, Ministry of Finance [17]. The incidence risk (IR) was calculated by taking into account the number of new cases who got anthrax infection in a projected population from 2012 census per 100,000 population in each region of the Tanzania mainland for a period of 2013–2016. The time period experienced by members of the population during which events of anthrax outbreaks occurred was also considered (Table 1).

**Table 1:** Spatial distribution of reported anthrax cases across various species in Tanzania Mainland, 2013 to 2016, based on the human and livestock National Surveillance Systems

Region	Estimated populations, 2012 Census (million)				Reported Cases and (Deaths) 2013–2016	Livestock deaths, 2013–2016			eIDSR (Human Cases) 2013–2016	Human (Incidence risk per 100,000 Pop)
	Human	Bovine	Caprine	Ovine	Human	Bovine	Caprine	Ovine		
Dodoma	2.08	1.5	1.0	0.26	0 (0)	23	39	87	0	0.00
Arusha	1.7	1.6	1.9	0.84	134 (8)	87	23	8	96	7.88
Kilimanjaro	1.64	0.65	0.69	0.25	109 (2)	17	35	26	38	6.64
Tanga	2.05	0.77	0.82	0.22	0 (0)	27	34	32	x	0.00
Morogoro	2.22	0.88	0.49	0.13	10 (0)	23	34	54	x	0.45
Pwani	1.10	0.54	0.19	0.04	0 (0)	7	32	32	x	0.00
Dar es Salaam	4.36	0.27	0.16	0.02	22 (6)	9	0	5	6	0.50
Lindi	0.86	0.26	0.10	0.01	0 (0)	7	8	9	x	0.00
Mtwara	1.27	0.17	0.23	0.02	14 (0)	28	4	12	x	1.10
Ruvuma	1.38	0.47	0.32	0.03	0 (0)	0	0	0	x	0.00
Iringa	0.94	0.66	0.20	0.04	0 (0)	0	0	0	x	0.00
Mbeya	2.71	1.45	0.56	0.08	2 (0)	16	2	0	x	0.07
Singida	1.37	1.37	0.83	0.29	6 (0)	12	31	21	0	0.43
Tabora	2.29	2.23	0.95	0.27	4 (0)	23	12	5	x	0.17
Rukwa	1.00	0.64	0.23	0.04	0 (0)	9	2	0	x	0.00
Kigoma	2.13	0.51	0.26	0.05	2 (0)	1	5	1	x	0.09
Shinyanga	1.53	1.30	0.62	0.20	0 (0)	21	13	4	x	0.00
Kagera	2.46	0.85	0.73	0.08	0 (0)	12	2	0	0	0.00
Mwanza	2.77	1.33	0.57	0.13	12 (0)	27	19	4	0	0.43
Mara	1.74	1.65	0.76	0.34	22 (8)	12	3	0	2	1.26
Manyara	1.43	1.81	1.54	0.58	8 (0)	26	13	4	1	0.55
Njombe	0.70	0.27	0.11	0.02	0 (0)	0	0	0	x	0.00
Katavi	0.56	0.36	0.18	0.03	0 (0)	12	3	0	x	0.00
Simiyu	1.58	1.60	0.93	0.39	0 (0)	2	0	0	x	0.00
Geita	1.74	0.82	0.43	0.05	0 (0)	0	0	0	0	0.00
Total	43.63	23.97	14.91	4.39	345 (24)	401	314	304	142	

Reported cases through the electronic system (eIDSR) and the reported human’s anthrax Incidence risk over the period of 2013–16 is also given

### Follow-up in hotspot areas in Arusha and Kilimanjaro regions

For the follow-up in Arusha and Kilimanjaro regions, we used data obtained from the randomly selected health facilities of the identified hotspot districts, Tanzania Wildlife Research Institute (TAWIRI) Serengeti National Park, Tanzania Veterinary Laboratory Agency (TVLA), District Veterinary Offices, Livestock Field Offices at the Ward and Village Levels and District Medical Offices. We conducted a comprehensive retrospective review of anthrax outbreak records in the human, livestock and wildlife health sectors of Northern Tanzania for the period of 2006 to 2016.

### Review at the health facility and animal diagnostic centres

Data reviews were carried out using the health management information systems (HMIS) booklets for both in–patients and out-patients at the health facility level. We also captured formal and informal meeting minutes, internal memos and official outbreak notification letters, and raw data in the form of typed or handwritten reports, tables and spreadsheets from the district medical and veterinary offices. Moreover, animal (livestock and wildlife) anthrax data were reviewed from the laboratory units of TVLA in Arusha, and TAWIRI in Serengeti National Park. We conducted this review in a period of two months, early October to late November 2016.

### Data management and analyses

We compiled the national datasets for humans and livestock into separate Excel® sheets. Cross-tables were obtained using Pivot tables in Excel, supported with tables generated in the statistical software Stata (Stata14/ SE, StataCorp, College Station, TX). We also entered follow-up data from Arusha and Kilimanjaro into Excel®. We classified the recorded human anthrax cases according to the name of the region, district, village/area of residence, health facility, sex, age, and date of anthrax diagnosis, and the outcome of treatment. We also used Excel® to create a trend (with computer generated moving average) of anthrax outbreaks from hotspot districts for the period of 2006–2016. As a means of data quality control, we excluded cases without proper records (as listed above) in the database. For livestock and wildlife diagnostic laboratory data the spreadsheet captured the name of the region, district, date of specimen collection, nature of specimen submitted, animal species, kind of laboratory tests and test results. All of this information was reviewed from the registers at TVLA in Arusha and TAWIRI in Serengeti. However, we omitted from the database any suspect animal cases without clear information on the date of specimen submission to TVLA. We entered all data into an Excel® spreadsheet, after establishing the Excel® databases, we created a map to indicate the locations of human cases by using the Quantum Geographical Information System (QGIS) software (http://www.qgis.org/en/site/forusers/ index.html). We cross-tabulated for age, sex, the location of human and animal cases, date of symptom onset, form of anthrax, final treatment outcome of human cases, and laboratory results across the species, over all seasons.

## Results

### Anthrax in humans

**Fig. 1:**
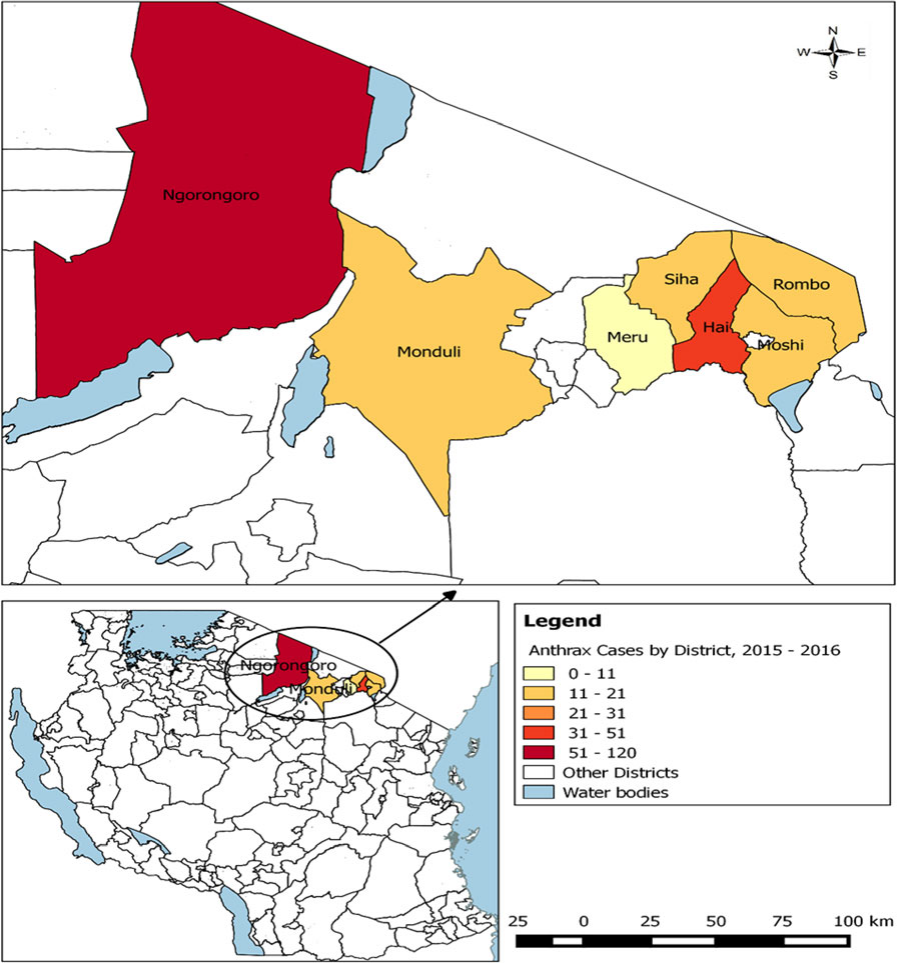
Map indicating the magnitude of human anthrax cases in the hotspot districts of Arusha and Kilimanjaro regions for the period of 2015 - October 2016

**Fig. 2:**
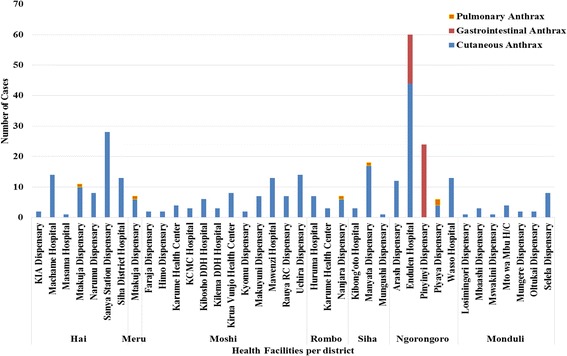
Distribution of clinical forms of human anthrax cases per health facility in hotspot districts of Northern Tanzania, 2006 to 2016

**Fig. 3:**
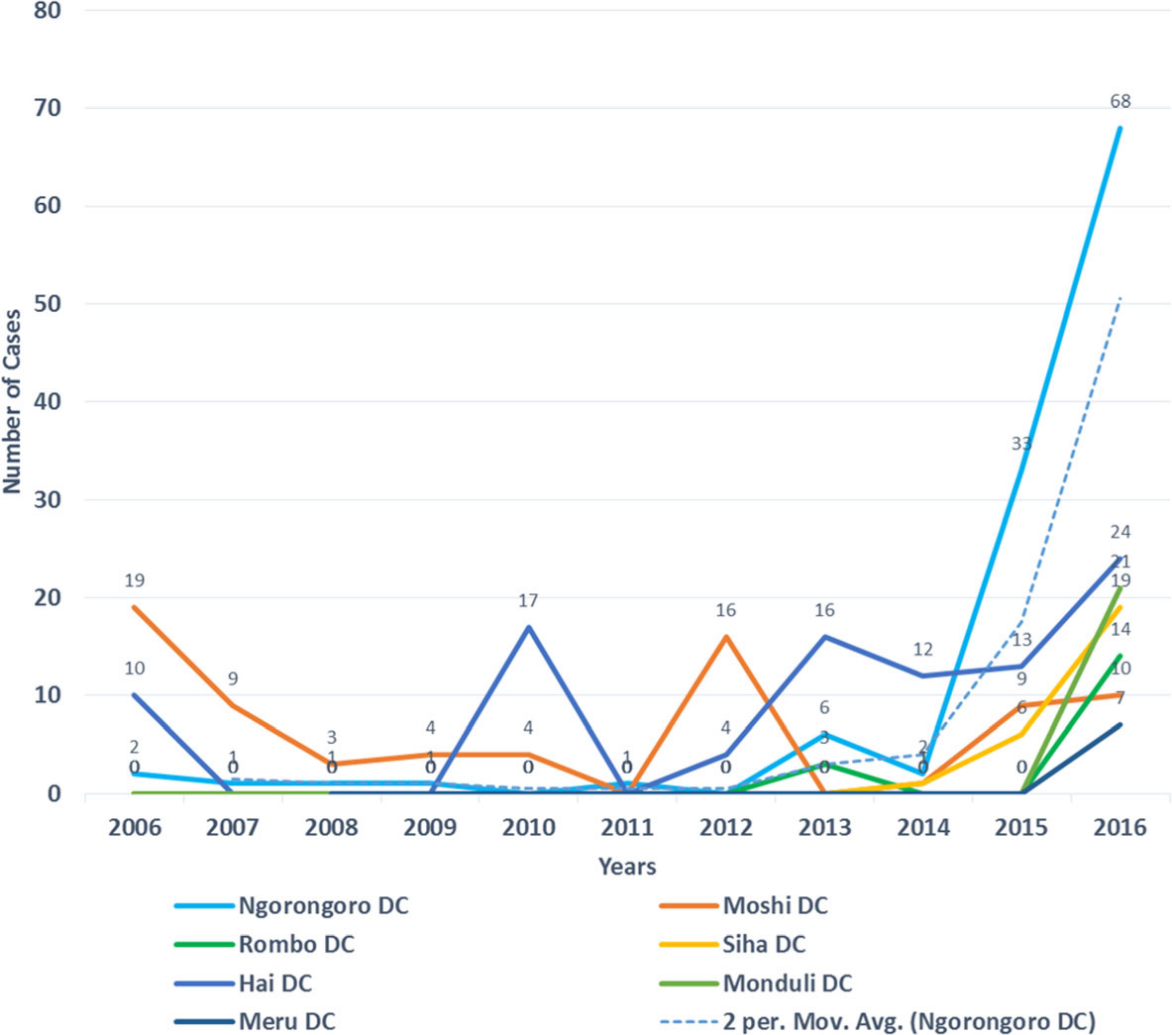
The trend of human anthrax cases from the hotspot districts of Arusha and Kilimanjaro regions, 2006 to 2016

We found that the reported human anthrax incidence risk over 2013–16 per 100,000 population was much higher in Arusha region (7.88/100,000) followed by Kilimanjaro region (6.64/100,000) than any other regions of the Tanzania Mainland (Table 1), identifying these regions as hotspots for anthrax. Records from selected health facilities showed that there were 187 human anthrax cases (57%) in Kilimanjaro and 143 (43%) in Arusha region for the period 2006–2016 (Table 2). Figure 1 indicates the spatial distribution and magnitude of anthrax cases in humans, while Fig. 2 shows the distribution of anthrax forms of human cases over the different health facilities. The majority (284/330, 86.1%) of all human anthrax cases reviewed at the selected health facilities were of the cutaneous form. A majority of reported human anthrax cases was in males, (215/330, 65.2%) compared with females. Figure 3 shows the trends of human anthrax cases in Arusha and Kilimanjaro regions for 2006–2016, illustrating an increasing trend with the highest number of 163/330 (49.4%) cases in 2016 showing 2 cases per moving average in Ngorongoro district in a time series of ten years.

**Table 2:** Distribution of human anthrax cases in study hotspot districts, Arusha and Kilimanjaro regions from 2006 to 2016

Regions	Districts	Number of Cases (% of Cases)
Arusha	Ngorongoro	115 (80)
	Meru	7 (5)
	Monduli	21 (15)
Total		143
Kilimanjaro	Moshi rural	71 (38)
	Hai	77 (41)
	Rombo	17 (9)
	Siha	22 (12)
Total		187

We found that there were human anthrax cases in record books since 2006 and beyond despite the disease not being included in the HMIS and IDSR reporting forms at that time. Overall, the Ngorongoro district reported more human anthrax cases, 115 (80%) compared to other districts of Arusha and Kilimanjaro regions. The Endulen Hospital reported more human anthrax cases (60/115, 52.2%) compared to other health facilities in Ngorongoro district in the period of review.

We further found a gross under-reporting of the electronic surveillance system in Arusha and Kilimanjaro regions. For instance, Arusha region reported 96 anthrax human cases through the electronic surveillance system from all the districts compared to 134 human anthrax cases obtained from health facility’s record review in hostspot districts only (Ngorongoro, Meru and Monduli) in the period of 2013 to 2016. Similarly, a total of 109 anthrax human cases were revealed following the health facility’s record review from hotspot districts only of Kilimanjaro region (Hai, Moshi rural, Siha, and Rombo) compared to 38 human cases reported by the electronic system from all districts in the region during the same period.

### Anthrax in livestock and wildlife

**Fig. 4:**
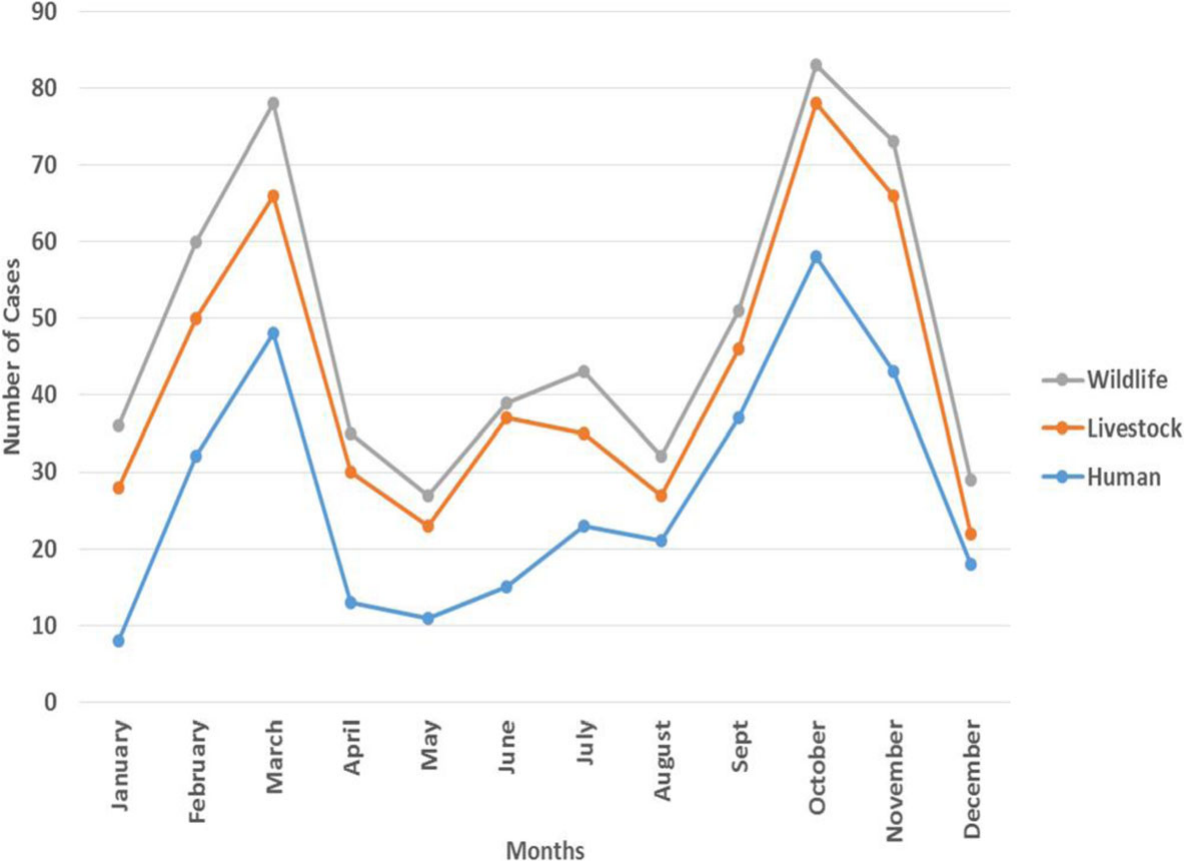
Monthly distribution of anthrax cases from all 3 health sectors (human, livestock and wildlife) in selected regions of Northern Tanzania, 2006–2016

From 2006 to 2016, TVLA received a total of 161 specimens from different livestock and wildlife species for laboratory analysis (Table 3). Most of the submitted specimens came from bovine (106/161, 66%). A total of 103 specimens (64%) tested positive for *B. anthracis,* and 68 (66%) of the positive specimens came from bovines, followed by caprine (18/103, 17%). In the same period, a total of 57 wildlife specimens obtained from active surveillance done in the Serengeti ecosystem were tested for anthrax at TAWIRI Serengeti laboratory. Of these 18 (32%) were positive for anthrax of which most of them came from African buffalo (12/18, 67%), (Table 4). Anthrax outbreaks have occurred across human, livestock and wildlife populations with peaks of outbreaks in the months of March and September through November and this corresponds to specific environmental conditions (Fig. 4).

**Table 3:** Summary of livestock species tested for *B. anthracis* at TVLA – Arusha, 2006 to 2016

Species Screened	Samples tested	*B. anthracis* (Positive)	% Samples testing positive
Bovine	106	68	66.
Caprine	23	18	17
Ovine	8	7	7
Swine	5	3	3
Wildlife trophies^a^	19	7	7
Total	161	103	

^a^Wildlife trophies: for the purpose of this review, means a group of unique wild animals whose parts of their body like horns, skin and skull are used for decorations like Waterbuck and Topi.

**Table 4:** Summary of wildlife species tested for *B. anthracis* at TAWIRI Serengeti laboratory in Serengeti National Park, 2006 to 2016

Species Screened	Samples tested	*B. anthracis* (Positive)^a^	% of Samples testing positive
African Buffalo	28	12	67
Elephant	2	2	11
Wildebeest	5	0	0.0
Black Rhino	1	1	6
Hippo	1	0	0.0
Giraffe	1	1	6
Horse	2	0	0.0
Zebra	11	1	6
Lion	1	0	0.0
Wildlife Trophies	5	1	6
Total	57	18	

^a^Using a microscopy test: Positive *B. anthracis* was obtained by staining a dry fixed blood smear with polychrome methylene blue. A typical morphology of the bacilli was observed to be gram positive, thick, long with square or truncated and swollen ends with characteristic ‘bamboo stick’ appearance

Generally, it was found that, the diagnostic capacity for anthrax in human, livestock and wildlife sectors was inefficient in the hotspot districts. This is because there were only two diagnostic centres for anthrax in Northern Tanzania, i.e. TVLA and TAWIRI Serengeti Centre for management of animal (livestock and wildlife) specimens. Human specimens especially skin lesion swabs were tested by Gram or Methylene blue staining techniques for anthrax in some of the selected health facilities. Other selected health facilities were managing anthrax cases clinically as they did not have any diagnostic capacity in place. No selected health facilities were found with advanced diagnostic capacity for anthrax like culture and PCR techniques.

## Discussion

This study has revealed that there is a close temporal correlation between the occurrence of anthrax outbreaks in animals (livestock and wildlife) and humans in the Arusha/ Kilimanjaro ecosystems. It might be attributed to the ongoing interactions between humans and animals such as types of husbandry, humans looking for food (meat and milk) and other livelihood issues like the use of animal skin as bedding materials, a common practice in the pastoral community.

We also, found more cases occurring in the dry season starting from September through November which might be a facilitating factor for anthrax transmission in animals and then into humans. The observed seasonal occurrences of anthrax show that climate-related factors (precipitation and ambient temperature) play a crucial role in triggering outbreaks, although there are variations between locations and therefore contributing factors are debated [9]. Some African countries, like this study have been reporting anthrax outbreaks at the end of dry seasons which indicates that over grazing, nutritional stress and congregation of animals at watering points might propagate the disease transmission [18, 19]. Also, animals tend to assemble themselves in certain places when there is pasture shortage, increasing chances of occurrences of anthrax [20].

Furthermore, water bodies may collect and accumulate spores in “storage areas” [21]. As water storage points are the last locations to hold water during dry seasons, these are the dangerous areas where animals tend to acquire the infection through drinking spore-contaminated water [22]. Our data demonstrated a seasonal pattern of anthrax outbreaks in northern Tanzania with peaks of outbreaks in humans, livestock and wildlife during March (start of long rain season) and September through November (end of dry season) each year in the last decade. This shows a high potential for anthrax infection in the human-livestock and wildlife interface areas of northern Tanzania [12], representing critical information to decision makers that they will need to set up preventive measures.

These measures could include strategic vaccination of livestock against anthrax, distribution of human antibiotic prophylactics to hotspot areas, and health education to high risk communities a few months before the expected time of anthrax outbreaks. Nevertheless, effective anthrax control depends on ensuring that the disease is controlled in livestock through routine targeted vaccination which may automatically control the problem in humans [20]. Restriction of free movement of healthy livestock during outbreak periods and safe disposal of dead animals (in a pit of six feet deep added with 10% formalin poured on top of the carcasses). This should be followed by soil decontamination on the area where the carcass is laying and removal of bloody soil, so it can help to prevent further occurrences of the disease [23].

We further found that men were at higher risk of acquiring anthrax (65.2%) than women, which might be due to slaughtering and handling the meat from the carcass of dead or slaughtered sick animals without inspection by a designated livestock officer. This is more likely to be the route of exposure for many of the cutaneous anthrax cases we found in this review [24]. More often they also eat meat while grazing their animals in the wilderness, only bringing home any remaining meat and offal for the wives and children. Moreover, men are the decision makers of the family who also dictates whether women and children should go to the hospital when they fall sick. This might impact on the health seeking behaviour of women creating a false representation in hospital registers regardless of the true disease status. Effective clinical management of zoonotic diseases depends on various factors including health seeking behaviour of individuals [16].

Pastoralists handle sick animals before dying and dress the carcasses after death [25]. Also, extensive handling of meat at different stages of preparations with direct skin contact with anthrax infected materials. It is a risk for contracting the infection and entry of *B. anthracis* into the human skin abrasions and can cause the cutaneous form of anthrax which we mostly found during this review. The cutaneous forms of anthrax are easily diagnosed clinically in health facilities and in laboratories by performing a Giemsa or Methylene blue staining on the discharges from lesions to detect the presence of *B. anthracis*. Other forms of anthrax like gastrointestinal and pulmonary are not as easily diagnosed at most of the existing health facilities in the anthrax hotspot districts in Tanzania.

High numbers of recorded human anthrax cases (Table 1) may partly be due to many patients that report at the specified health facility and good systems of recording patients in the health management information system (HMIS) outpatient & in-patient department booklets. In a few instances, some health facilities had no HMIS booklets which may account for no or a low number of reviewed human anthrax cases. For example, the Magaiduru dispensary in Ngorongoro district had no HMIS booklets to keep records of human anthrax cases regardless of some verbal information on the presence of human anthrax suspected cases in the village they serve. The same applies for IDSR reporting forms that in past years anthrax was not one of the IDSR priority diseases. Therefore some facilities did not bother to report the disease until 2013 when a revised National IDSR Guidelines included anthrax as one of the immediately reportable diseases. However, in Hai district they historically improvised a slot on the reporting forms for capturing anthrax cases in the infectious diseases weekly ending (IDWE) reporting forms which accounts for a high number of anthrax cases in Hai district compared to other hotspot districts of Kilimanjaro region. Overall, Arusha region has reported more anthrax human cases in a time series of the last ten years and Ngorongoro district having more anthrax human cases compared to other districts. This might be contributed by the pastoral communities living in close proximity with wildlife conservation areas and facilitating disease transmission between livestock and wildlife animals and then to humans.

Anthrax outbreaks cause substantial economic losses through livestock and wildlife losses, the cost of laboratory reagents and carcass disposal (burial or incineration). Therefore investment in the control of this disease is inevitable [26]. Response to these outbreaks requires joint collaborative efforts of the Ministries responsible for human health, livestock, and wildlife services. However, one of the biggest challenges in the control of zoonotic diseases is the current lack of joint approaches for responding to disease outbreaks. Therefore, there is a need for the creation of joint response action plans with combined technologies and infrastructures from both public health and veterinary professionals including sociologists, and ecologists to initiate approaches to contain zoonotic diseases [27]. Worldwide, a One Health approach is a call to action for the establishment of closer professional interactions, collaborations, capacity building and research opportunities across the science professionals and related disciplines to improve the health status of humans, livestock, wildlife, and the environment [28]. In Tanzania, one of the challenges for initiating the joint surveillance system under a One Health approach would be lack of compatible surveillance systems between the ministries responsible for human health, livestock and wildlife. Nonetheless, the right opportunity is the existence of a strong IDSR system within human health sector which can be improved and expanded to cover a harmonized list of priority zoonotic diseases  in a ‘One Health’ approach.

In countries like Kenya the five diseases identified as top priority zoonotic diseases are anthrax, trypanosomiasis/HAT, rabies, brucellosis and Rift Valley Fever (RVF) in descending order [29]. This highlights the importance of prioritizing zoonotic diseases in Tanzania, as well as presenting opportunities to focus on diseases with the greatest local public health burden and not focus only on diseases that have greater global attention [29]. Most often, authorities start looking for the disease in livestock and take appropriate actions only after they report human cases and deaths in that particular area. When disease surveillance and control take this approach, humans essentially serve as a sentinel species (human illness and death) act as proxy indicators of disease prevention and control in livestock [12].

We also observed that there is poor anthrax diagnostic capacity, not only in the hotspot districts in northern Tanzania, but the entire country. The only routine diagnostic techniques performed at TVLA in Arusha and TAWIRI in Serengeti National Park are either Giemsa or Methyline blue staining of fixed blood smear from either humans or animals (livestock and wildlife). They also test swabs collected from skin lesions of human anthrax suspected cases by the same technique at some health facilities of the hotspot districts in Northern Tanzania. The Ministry of Livestock and Fisheries headquarters has a laboratory (Biosafety Level 2 Laboratory) with a capacity for diagnostic polymerase chain reaction (PCR) for anthrax, but is located in Dar es Salaam about 700 Km away from the hotspot areas. This hampers the diagnosis of other forms like pulmonary and gastrointestinal anthrax, which are currently managed clinically at respective health facilities and may be confused with so many other diseases with a potential of causing pneumonia and/or bloody diarrhoea within the hotspot areas. There is a concern for biosafety in clinical laboratories; the requirements vary in different countries. We consider Biosafety Cabinet level 2 appropriate for clinical laboratory analysis, while biosafety level 3 is more suitable for research related studies involving spore suspensions in liquids formulation or large-scale cultures [30]. We would therefore recommend an animal US snap test for anthrax, which needs a specimen from a dead animal (tissue or blood) and obtain results within a short time. Similar tests can be pursued for humans after having good response in the animal sector in terms of supportive political will, user acceptance and accessibility of the relevant technology and gadgets.

In most instances, many anthrax outbreaks are mismanaged, particularly in rural areas, where it is unlikely to have been adequately diagnosed, reported on time and forwarded to the central levels for rapid response. Under-reporting of the IDSR priority diseases (including anthrax) through the electronic surveillance system was revealed in Arusha and Kilimanjaro regions. Therefore, anthrax cases detailed in this study include only those that were recorded, communicated and reviewed in the surveillance systems of Tanzania. It only provides an estimate of the magnitude of the disease which could be a significant under - estimate of the disease burden. Furthermore, the collected anthrax data would assist in future ecological niche modelling in order to map for areas where anthrax outbreaks are more likely to be occurring. This may be a tool for optimization of control measures and improving epidemiologic knowledge of this disease in Tanzania. The causality of various potential risk factors for anthrax transmission in the affected communities of northern Tanzania could also be tested with appropriately designed future observational studies.

## Conclusion

The findings of this study are critical for consideration by respective authorities for setting up prevention and control measures of anthrax outbreaks in the human, livestock and wildlife sectors within Tanzania. There is a gross under-reporting of anthrax cases in existing human and animal surveillance systems, which can be an obstacle for estimating the real burden of anthrax in the hotspot districts. We also noticed that people living in the marginalised communities like the Maasai remain at high risk of contracting anthrax infection given their ties to cultural practices of handling and consuming dead animals and their products. Moreover, repeated occurrences of anthrax in livestock, wildlife, and humans suggest for strengthening links and promoting inter–disciplinary and multi-sectoral collaboration to enhance the improved prevention and control measures for anthrax outbreaks in a One Health approach.

## Abbreviations

AHIS: Animal Health Information System
eIDSR: electronic Integrated Diseases Surveillance and Response System
GIS: Geographical Information System
GPS: Global Positioning System
HAT: Human African Trypanosomiasis
HMIS: Health Management Information System
IDSR: Integrated Diseases Surveillance and Response
IHR: International Health Regulations
IPD: inpatient Department
MNRT: Ministry of Natural Resources and Tourism
NIMR: National Institute for Medical Research
OPD: Outpatient Department
PCR: Polymerase Chain Reaction
PoE: Point of Entry
QGIS: Quantum Geographical Information System
RVF: Rift Valley Fever
TAWIRI: Tanzania Wildlife Research Institute
TVLA: Tanzania Veterinary Laboratory Agency
WHO: World Health Organization
